# Clinico-Histopathological and Immunohistochemical Study of Ruminant's Cutaneous Papillomavirus in Iraq

**DOI:** 10.1155/2020/5691974

**Published:** 2020-02-21

**Authors:** Karima A. AL- Salihi, Ahmed H. Al-Dabhawi, Ali Abbass Ajeel, Ibrahim A. Erzuki, Tho Alfiqar H. Ali

**Affiliations:** ^1^Head of Department of Veterinary Internal Medicine, College of Veterinary Medicine, Al-Muthanna University, Samawah, Iraq; ^2^The Dean of Faculty of Veterinary Medicine, Department of Veterinary Pathology, Faculty of Veterinary Medicine, University of Kufa, Najaf, Iraq; ^3^Head of Department of Veterinary Surgery, College of Veterinary Medicine, Al-Muthanna University, Samawah, Iraq; ^4^Al-Muthanna Veterinary Hospital, Al-Muthanna Governorate, Iraq

## Abstract

The papilloma viruses are constituted of double-stranded DNA and are a more common lesion in ruminant's skin in Iraq. The p53 tumor suppressor protein reveals an essential role in cell cycle control. This study intends to investigate the clinical, histopathological, and immunohistochemical features of cutaneous papilloma in ruminants in Iraq. Samples had been collected from a total of 10 animals (three cattle, three goats, and four sheep) with multiple papillomatosis lesions. The samples were processed for histopathological and immunohistochemical techniques. Clinically, the lesions appeared as multiple various sizes (0.5–11 cm), cauliflower exophytic masses on different parts of the animal's body. The histopathological features of the epidermis granular layer revealed perinuclear vacuolation (koilocytosis) accompanied by various degrees of hypergranulosis, hyperkeratosis, acanthosis, orthokeratosis, and parakeratosis. Strong positive reaction for papillomavirus antigen was seen in both epidermal basal and granular layers in the immunohistochemical investigation (IHC). Moreover, all papilloma lesions revealed an intense positive p53 reaction in cytoplasmic and perinuclear of the basal and parabasal layers. In conclusion, this study described the papillomavirus lesions in bovine, ovine, and caprine animals, which were found in different parts areas of the affected animals. All lesions show similar histopathological features with minor variations. PV antigen and p53 protein expression showed positive results in immunohistochemistry that can be used as diagnostic markers for ruminant's papilloma.

## 1. Introduction

Bovine viral papillomas, also commonly known as warts, are caused by the bovine papilloma virus (BPV) that result in the proliferation of the skin and development of verruciform lesions [1, 2]. BPV is primarily self-limiting, but warts may be removed either for cosmetic reasons or if it is irritating the animal (e.g., near the eyes). Papilloma viruses produce both benign and malignant tumors in ruminant, like cutaneous papilloma, oesophageal, and urinary bladder cancer and benign fibroplasia and cause significant economic losses [1, 2]. The virus prefers the stratified squamous epithelia of warm-blooded animals, for example, bovine. Similarly, only the  horses and other equids revealed cross-species infection that is reported in the literature [3]. Meanwhile, globally, viral infection occurs in all species of animals and is highly species-specific [3]. The bovine papillomavirus contains circa eight kilobases genome of double-stranded circular DNA [4] and belongs to papovavirus family and are constituted of icosahedral, nonenveloped symmetrical DNA that measures 50–55 nm in diameter.

Hitherto, scientists have determined twelve different species-specific virus serotypes. The fibro-papillomaviruses related to the genus Deltapapillomavirus are called BPV1 and 2 and have an attraction for the dermal and epithelial tissues [5]. While BPV-3, 4, 6, 9, and 10 are classified within Xipapillomavirus genus and epitheliotropic. However, BPV-5, 7, and 8 cause cutaneous fibro- and epithelial papillomas and belong to the genus Papillomavirus [6]. Papillomaviruses are host-specific in natural infection. However, BPV-1 and 2 can induce fibroblastic tumor in equine [3]. There is no clinical risk from cutaneous papillomas.

Nevertheless, specific genetic and environmental factors may lead to malignant transformations. Papillomaviruses infection can result in significant economic animal losses due to reduction in milk and meat production and poor hide value. Ruminant papilloma can occur at any age. However, animals of less than two years' age are more susceptible [4]. Cattle are the principal natural carriers of papillomavirus that enters the host body via body scratches or injuries by direct or indirect contact. Moreover, malnutrition, hormonal imbalances, artificial insemination, long-term exposure to sunlight accompanied with immunodeficiency, and contaminated utensils such as milk machines and syringes are all factors that play an important role in the occurrence of the disease [7]. Clinical lesions appearing on the skin are a very important indicator for primary diagnosis that needs to be supported by histopathological features and electron microscopy investigation [8]. A large number of publications reported polymerase chain reaction as a necessary tool in the diagnosis of bovine papilloma using consensus primers (FAP59/FAP64 and My09/MY11), which was designed based on human papillomavirus genome encoded in L1, L2, E6, and E7 structural proteins [9]. The p53 (tumour suppressor protein) mediates several mechanisms in the cell, such as programmed death of the cell (apoptosis), cell cycle arrest, and the case of being old (senescence) that occur due to cellular tensions factors like oxidative damage, hypoxia, carcinogenic deregulation, DNA damage, integrity, and repair [10–14]. The nucleus p53 transfers and interrelates to the cell cytoplasm in the presence of severe stress and controls bcl-2 protein family that contributes to activation apoptosis due to the liberation of the proapoptotic protein. The p53 protein has an essential functional role in regulating the cell cycle, and absence of its suppressive mechanism contributes to the development of various human tumors, for example, squamous cell carcinoma, ameloblastoma [15, 16], and animal tumors, like BPV-induced tumors [11, 14]. Papillomavirus is considered as the most distributed carcinogenic virus related to ruminant (sheep, goat, and cattle) malignant and benign tumors in Iraq and afffects the economy significantly. A review of the literature revealed scarce publications concerning p53 protein expression and detection of papillomavirus in the affected ruminants in Iraq. Consequently, the current study emphasizes on the clinical and histopathological appearance of ruminant (bovine, ovine, and caprine) papillomavirus lesions and also on detecting the papillomavirus antigen in the cutaneous papilloma lesions and the expression of p53 protein using immunohistochemistry.

## 2. Materials and Methods

### 2.1. Animals and Sample Collection

**Figure 1:**
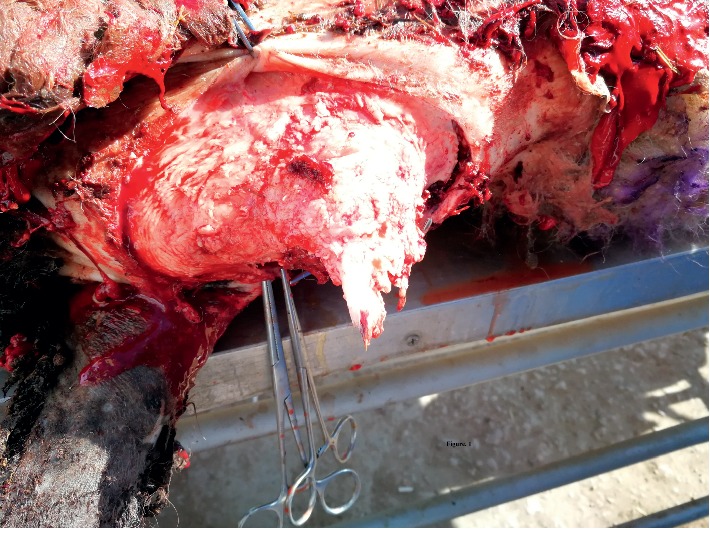
Papillomavirus lesion in sheep, surgical removal, and sample collection for histopathological and immunohistochemical investigations.

Ethical approval was issued for the current study by the ethical and research committee, Al-Muthanna University (2017/2018-10-Res.pro). Papilloma or fibropapilloma cutaneous lesions were collected from naturally affected ruminants in Al Muthanna province, Iraq (Table 1). The study included three cattle, three goats, and four sheep that were presented to the Al-Muthanna Veterinary Hospital from September 2017 to April 2018. A clinical examination was done on the affected animals that revealed a growth compatible with the classical growth of PV. All the animals included in this study showed multiple, cutaneous  tumours, localized anatomically in different areas of the animal's body (eyes, muzzle region, head, neck, back, testes, and udder) (Table 1). Lesions were removed surgically (Figure 1) according to routine procedures after disinfection with 70% alcohol. The border of the lesion's skin was surrounded with parallel cut, and then lesion mass (wart) was removed using a sterile scalpel and preserved in disposable containers. The samples were cut into small pieces for further investigations. Later, according to the conventional histological technique [17], the wart pieces fixed in 10% neutral-buffered formalin were embedded in paraffin wax and sectioned to a size of 4-5 *μ*m. The sections were stained with hematoxylin-eosin (H&E) and examined by using a light microscope. The diagnosis was done according to the rules suggested by [18] and depending on the histopathological appearance.

### 2.2. Immunohistochemistry

#### 2.2.1. Detection of PV Antigen

The immunohistochemical technique used for detection of papillomavirus was done according to the method that uses the ABC (streptavidin-biotin-peroxidase) technique [19]. Briefly, tissue sections (5 *µ*m) were dewaxed in xylene and hydrated by a graded alcohol's concentration. Then, H_2_O_2_ (3%) was added for 15 minutes to block the activity of endogenous peroxidase. Next, the sections were washed with PBS (pH 7.2), soaked in citrate buffer (pH 6.0), and kept in a microwave oven (800 Watt) for 10 minutes, for antigen recovery. All sections were washed with PBS, and the rabbit anti-papillomavirus antibody (DakoCytomation and Glostrup, Denmark) (1 : 1000 diluted) was added and incubated for 30 minutes. Then, the biotinylated secondary antibody (Histostain®-Plus kit, Cat No. AA85-9043) and streptavidin HRP (Histostain®-Plus kit) were added for 10 minutes at room temperature. Later, all sections were counterstained with Gill's hematoxylin. The primary antibody was excluded from the negative control section, and the sections were incubated with diluted normal serum from the species where the primary antibody was raised.

#### 2.2.2. Detection of p53 Expression

The streptavidin-biotin method (LSAB Kit, Dako) was used for immunostaining the papilloma sections and two normal skin samples. The paraffin sections (5 *µ*m) were deparaffinized in xylene and 100% ethanol, and then 0.3% hydrogen peroxide methanol solution was added for 20 minutes to block the activity of endogenous peroxidase. The antigen was recovered by heating in a microwave (700 Watt) for two cycles, each cycle for five minutes with sodium citrate (pH 6.00). Later on, all sections were allowed to cool for 10 minutes and rinsed with phosphate-buffered saline (pH 7.4/0.1 M). Nonspecific binding proteins were blocked by adding block serum (Dako) for 15 minutes, and mouse anti-p53 antibody (dilute to 1 : 50 in PBS) (NCL-p53-505, Novocastra) was added for one hour at room temperature in a humidified chamber. Then, the biotinylated secondary antibody was added for 20 minutes, and streptavidin conjugated to horseradish peroxidase was added after the washing step in PBS for 20 minutes at room temperature. Finally, the diaminobenzidine (DAB) (Dako) was added for five minutes to develop colour, and all sections were counterstained with Mayer's hematoxylin. The primary antibody was excluded or replaced with normal saline for negative control sections. Human  mammary cancer paraffin-embedded sections were used as a positive control for p53 protein expression. The immunoreactivity reaction was investigated on each sample in a blind method by two examiners, and the reaction was scored from no reaction to a very strong reaction.

## 3. Results

### 3.1. Clinical Signs and Gross Appearance

Bovine warts were found over all parts of the body of the affected animals. These cases revealed the most common, classical types of papillomas and were compatible with the bovine cutaneous lesions reported previously in the literature. Moreover, the age of the affected cattle varied from six months to five years.

**Figure 2:**
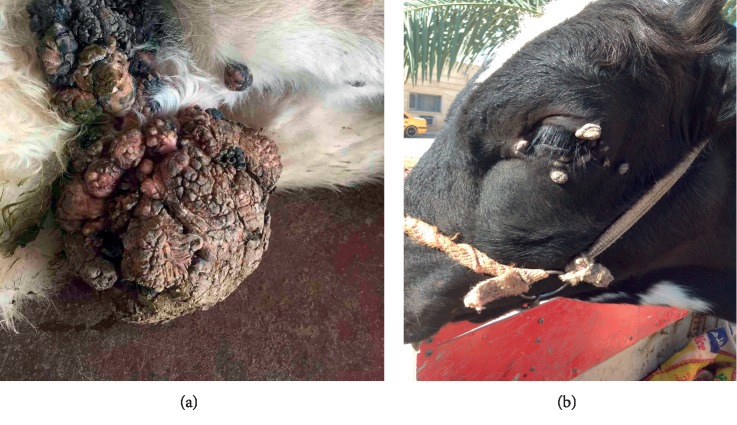
Papilloma virus lesions in the adult cow. (a) Multiple lesions on the abdominal wall. (b) Multiple lesions on the eyes.

**Figure 3:**
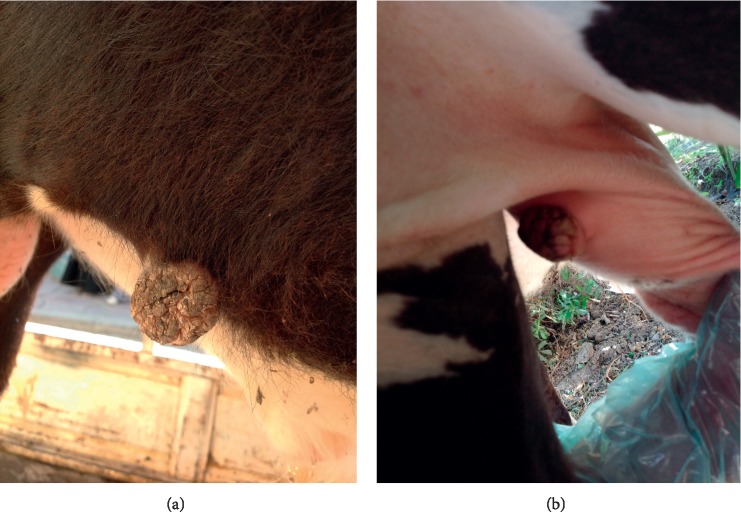
The lesions of papillomavirus in calf. (a) A single lesion on the abdominal wall. (b) A individual and sessile on the testes.

The disease occurred in both sexes (two females and one male). The lesion was located on the female external genital areas. While in the male, the lesions were found in the testes, face, around the eyes neck, shoulders, and perianal (Figures 2(a) and 2(b); Figures 3(a) and 3(b)). The lesions were variable in size with dry, horny, and verruciform shape.

**Figure 4:**
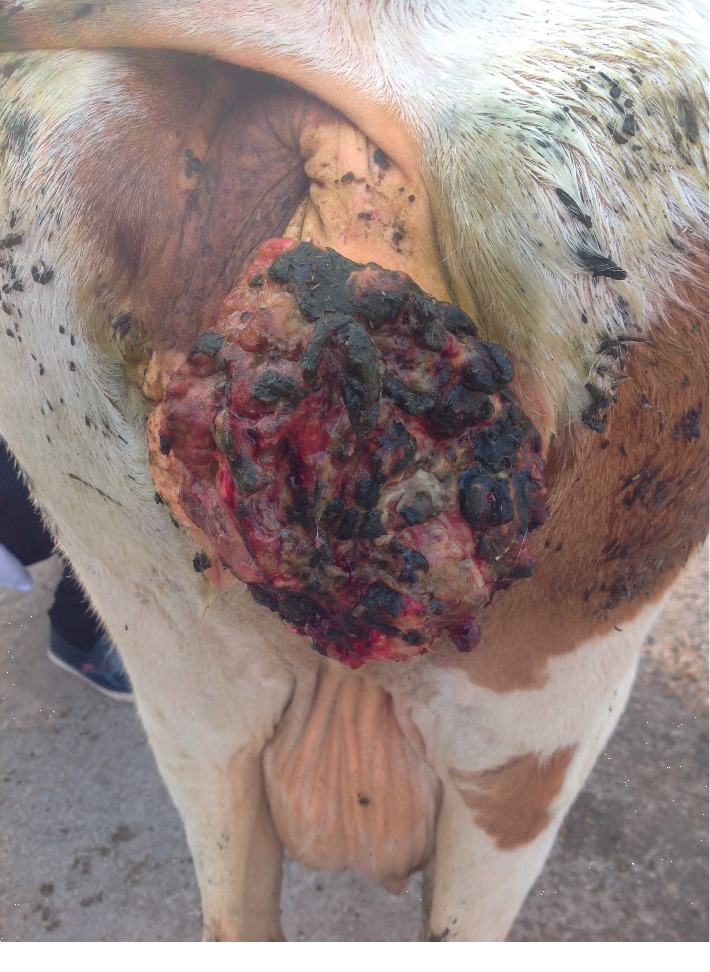
A huge papilloma lesion that appeared on the cow's external genital tract.

**Figure 5:**
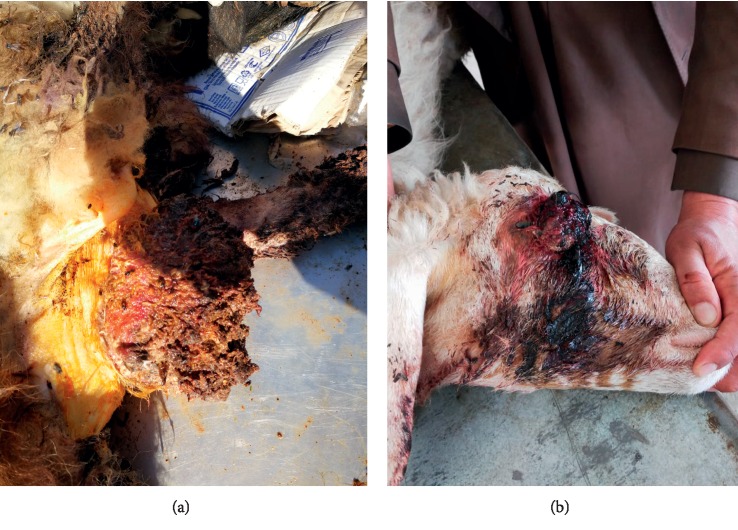
The papilloma lesions in the sheep. (a) The papilloma lesion found on the sternum. (b) The lesions on the eye.

The lesions did not regress spontaneously in all animals, and warts persisted for more than five to six months and two years in one case (according to a case history), with severe loss of body condition. In this, an extensive infected lesion oozing pus was seen on the genital area (on the vagina) of one cattle (Figure 4); moreover, single and sessile lesions were seen on the testes. Both males and females with genital warts (on the vulva and penis) suffered from difficult mating because of easy bleeding of large size lesions. All lesions were surgically removed, especially the large lesions (Table 1). In sheep, the lesions were recognized on the ears, lips, eyes, face, shoulder, thigh, chest, and sternum (Figures 5(a) and 5(b)).

**Figure 6:**
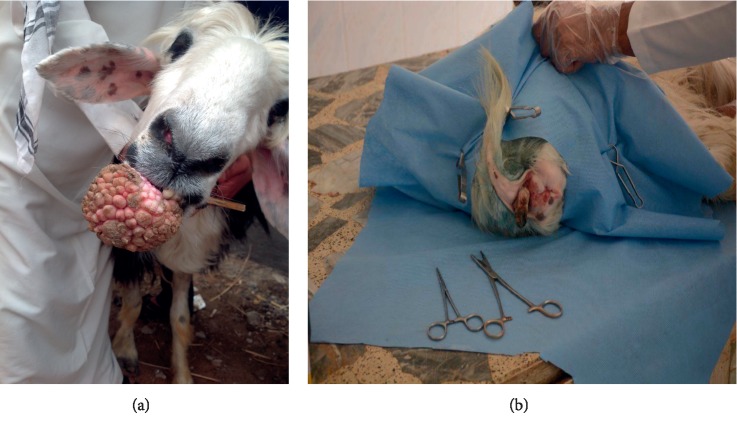
Papillomatosis of the goat. (a) The apple size fibropapilloma located on the lower lip. (b) A large lesion located on the perianal region.

Moreover, one sheep revealed extremely extended lesion on the sternum that reached the cartilage. Additionally, one ewe showed a complex, unhealed, and bled lesion on the thigh due to screwworm infestation after tough removal of the wart by the owner. The ewe became very emaciated.  In goats, one animal revealed a huge lesion, equal to the size of an apple, located on the lower lip with deformity of the mandible and loss of teeth. It caused interference with the prehension of food (Figure 6(a)). On the other hand, the lesions appeared on different body areas of another goat. Nonetheless, the lesions in the perianal region were large and affected the breeding of the animal (Figure 6(b)).

### 3.2. Histopathological Features

**Figure 7:**
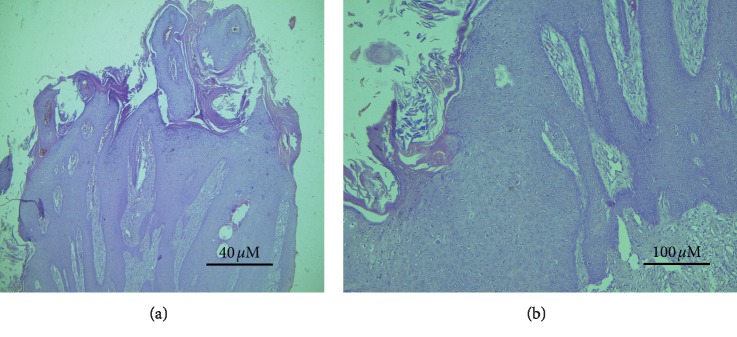
Acanthosis, superimposing stratum corneum, accompanied with finger-like projecting papillae and rete pegs grown downwards homogeneously. (a) (×4); (b) (×10), H&E (sheep section).

Microscopically, papillomas were covered by stratum corneum and acanthosis and appeared as mature finger-like prominent papillae accompanied by down-growing rete pegs (Figures 7(a) and 7(b)). All animals revealed irregular papillary projection and a varying degree of epidermis hyperplasia.

**Figure 8:**
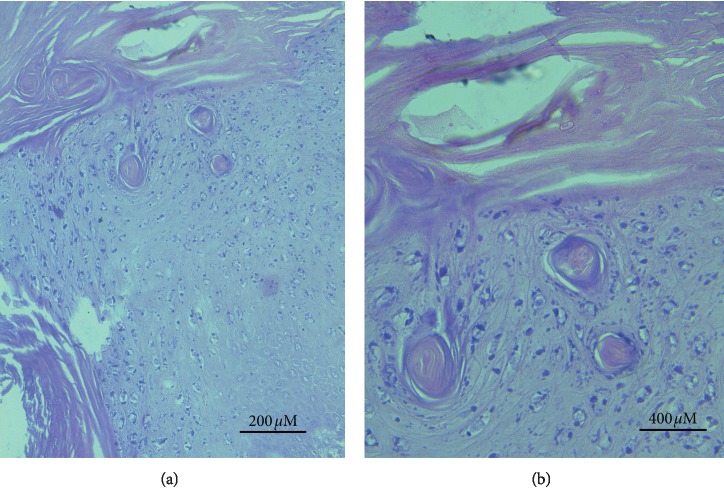
Bovine section with hyperplastic epidermal cellular layers, islands of connective tissue, and basket-wave-like hyperkeratosis accompanied with hyperplastic stratum spinosum. (a) (×20). (b) (×40), H&E.

Bovine cases had a diagnosis of a state where “papilloma” consisted of the moderate-to-extensive degree of cornification (hyperkeratosis), with basket-weave features. Moreover, hyperplastic stratum spinosum with many koilocytes, different degrees of parakeratosis, and the island of connective tissue surrounded by hyperplastic epidermal cell layers were also prominent (Figures 8(a) and 8(b)).

**Figure 9:**
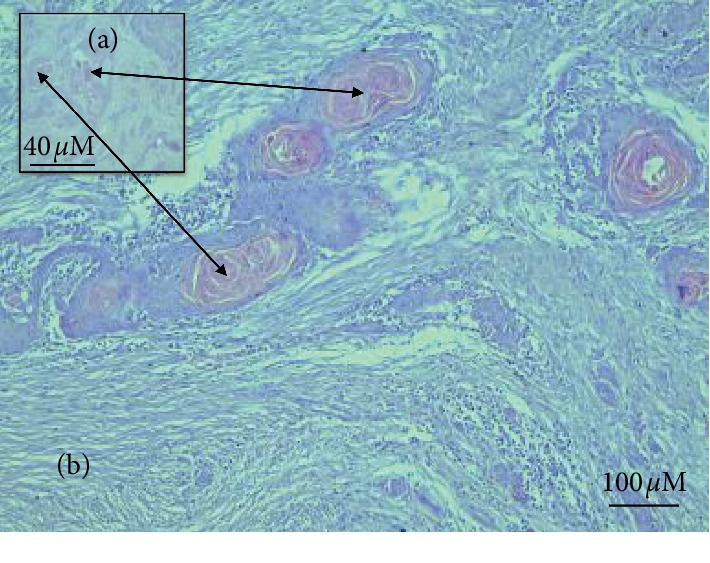
Invasive growth pattern and star-shaped large fibroblasts accompanied by severe proliferative process. (a) (×4); (b) (×10) (bovine section).

**Figure 10:**
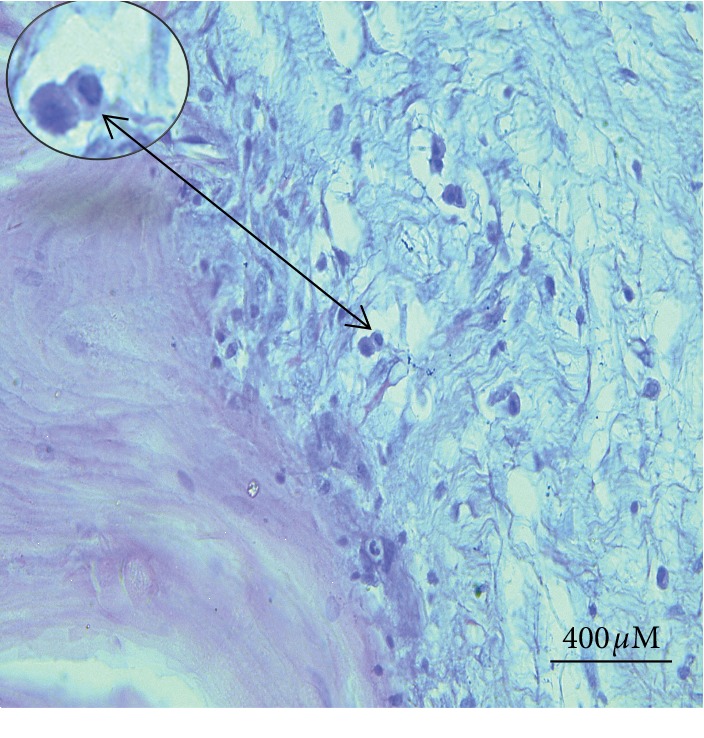
A moderate to severe mitotic activity with a divided hyperchromatic nucleus in the basal cell layer appeared clearly in papilloma lesions from the bovine section.

**Figure 11:**
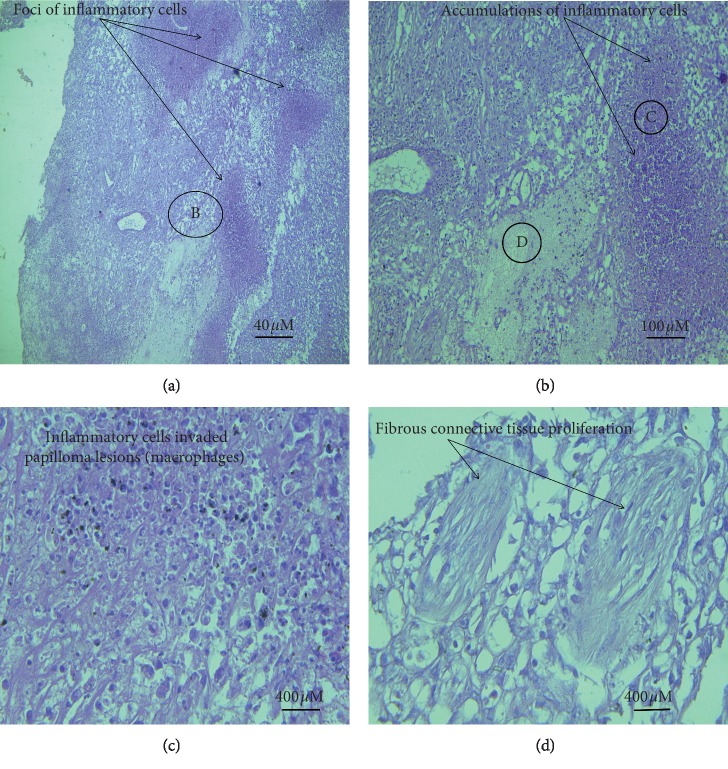
The accumulation of inflammatory cells, especially the macrophages. The inflammatory cells appear as multiple foci. (a) (×4). (b) (×10). (c) (×20). (d) The inflammatory cell infiltration and fibrous connective tissue in the bovine sections (×40).

The invasion was seen in the basal cell layer with hyperplastic activities characterized by hyperchromatic nuclei and moderate to severe mitotic activity. The neoplastic stromal tissue comprised large stellate shape fibroblasts and heavy fibrocellular proliferative changes that were found below the epidermis (Figures 9(a), 9(b), and 10). The histopathological sections prepared from the large external genital papilloma (oozing pus and easy-to-bleed) showed various types and a large number of inflammatory cells invading the area (Figures 11(a)–11(d)). The majority were 1macrophages and few polymorph nucleus cells.

**Figure 12:**
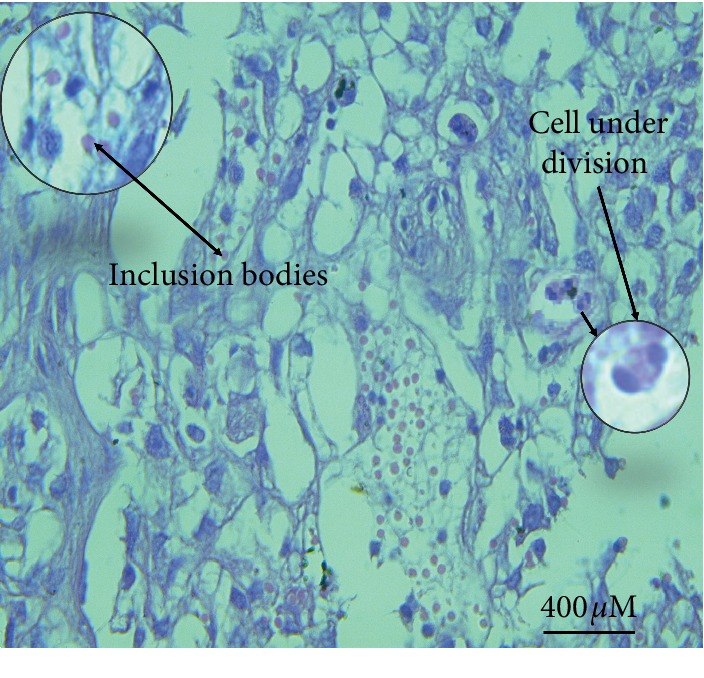
Intranuclear inclusions (10–15 *μ*m in diameter) frequently found as slightly basophilic to eosinophilic structures in the keratinocytes (×40, H&E) (sheep section).

In sheep and goats with fibropapilloma (exophytic) warts, the lesions revealed only scarce koilocytes in the upper layer of the stratum spinosum. The histopathological structures were similar in all cases except some revealed elongated rete pegs that extended and proliferated extensively toward fibrous stroma called endophytic fibropapilloma. Papilloma occult and fibroblastic papilloma were also seen in histopathological sections of some cases. Ballooning degeneration appeared in some cases in different stages accompanied by pleomorphic and profuse keratohyalin particles. Lesion sections prepared from cases with secondary infection revealed dermal and epidermal neutrophil infiltration. Rarely, some sections revealed free melanin particles in the dermal melanophages and subepidermal layers. Majority of cases showed slightly frequent basophilic to eosinophilic intranuclear inclusions measuring 10–15 *μ*m in diameter (Figure 12).

## 4. Results of Immunohistochemistry

**Figure 13:**
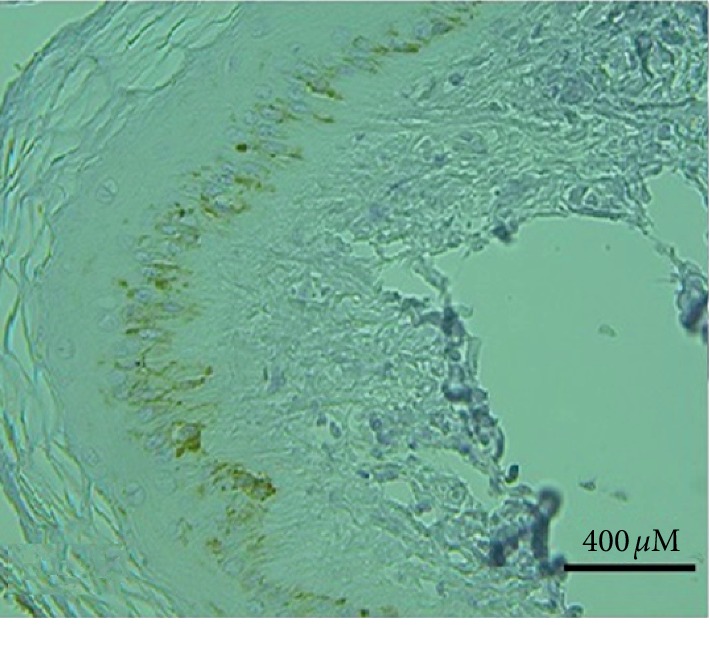
Bovine skin shows a positive reaction in the nucleus of epidermal basal and granular layers cells for papillomavirus antigen. The nuclear residues also show a strong positive reaction in the stratum corneum (×20, H&E).

**Figure 14:**
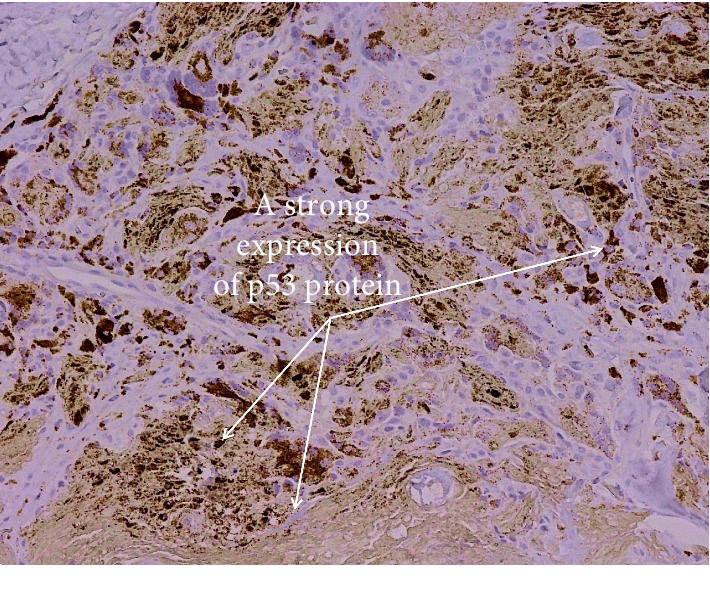
Bovine skin shows variable expression of papilloma lesion to p53 protein with a strong reaction in most sections (×40, H&E).

**Figure 15:**
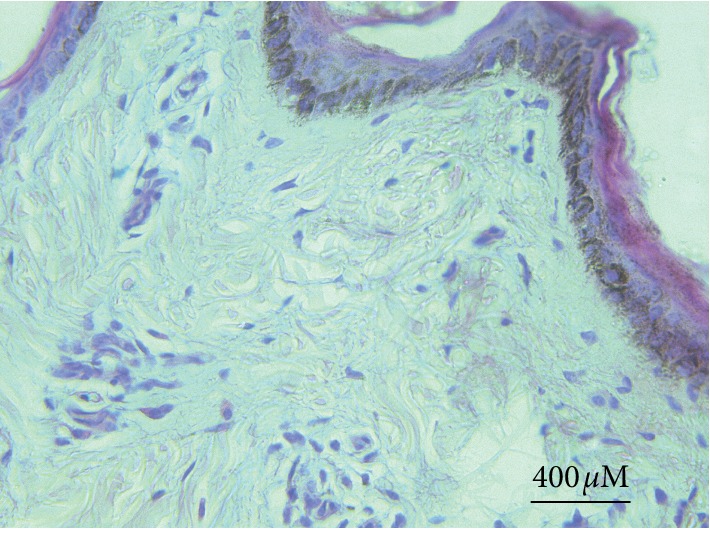
The normal bovine skin section revealed a negative p53 staining reaction.

Strong positive reaction for the papillomavirus antigen was seen in some cellular nucleus of the epidermis granular and basal layers (Figure 13). The nuclear residue also revealed strong positive reaction in minor hollows of the stratum corneum that existed due to the disappearance of the nuclei. Variable positive reactions (strong to weak) were seen in the cytoplasm with some nucleus positivity of the connective tissue and mesenchymal cell of the fibropapillomas. Irregular weak reactions were rarely seen in few vascular endothelial cells. P53 positive reaction was seen in all cases (papilloma and fibropapillomas samples). A strong reaction appeared in the cytoplasm and perinuclear of the stratum corneum, parabasal, and basal layers' cells (Figure 14). Also, few cells of granular and spinous layers revealed a strong reaction. In the spinous and granular layers, few cells with intense perinuclear staining were also observed. Meanwhile, sections prepared from normal skin showed negative p53 cytoplasmic staining (Figure 15).

## 5. Discussion

Papillomatosis stands as the most popular bovine viral skin disease, and warts or nonmalignant tumors are its clinical presentation. Bovine papillomaviruses are the causative agents which induce the disease in all ruminant age groups. However, middle age and young cattle less than two years are more affected [3]. The results of this study clinically investigated the papilloma lesions in ruminants (bovine, ovine, and caprine). The ages of infected animals were extended between 6 months and five years. The thorax, head area especially around the eyes, and neck were the most anatomical clinical presentation regions for the lesions. However, the lesions were located in other parts of the animal body in a few animals. These clinical presentations are in agreement with the previous observations reported by another researcher [20].

Moreover, Araldi [2] found that papillomatosis might be a severe problem of cattle herds if a large number (20–25%) of young cattle were infected [8]. The results of this study showed the detection of papillomatosis between a male calf presented with multiple body lesions and two females. The results of the macroscopic presentation and microscopic features of the lesions were compatible with the findings reported previously in [6].

A majority of wart lesions appeared grossly as verruciform lumps (warts), while the histopathological examination revealed marked epidermis hyperkeratosis and extended odd papillary projections into the dermis. The natural infection of bovine papillomaviruses is seldom accompanied by the presence of inclusion bodies in the skin lesions [20]. Nonetheless, numerous faintly basophilic to eosinophilic intranuclear inclusions were observed within keratinocytes and tumor cells in the results of this study.

These histopathological results are compatible with a previous study [21], that demonstrated the presence of intranuclear viral inclusion body and revealed a positive reaction for bovine papillomavirus 1 in the cells of the epidermis basal layer. Bovine papillomaviruses belong to the papovavirus family, and the cattle are considered as the natural host and carrier of the virus [21].

The histopathological observations seen in the current study are compatible with the results reported previously on bovine papillomavirus in the middle of Iraq by other researchers [22].

Various cellular markers use for identification of tumor proliferations. The proliferation level of tumor tissue cells can be investigated by microscopic estimation of nuclear antigen that is related to cell division and growth using the immunohistochemical tools. The results of immunohistochemical staining of the current study revealed few nuclei of epidermis basal and granular layers with intense positivity for the papillomavirus antigen. Moreover, nuclear remnants in the stratum corneum showed an abundantly positive reaction. All these results agree with other studies that approved extreme reaction for the papillomavirus antigen [19, 23].

Estimation of dermal neoplastic cell proliferation is investigated by different markers, and p53 is a valuable one. The observations of the immunohistochemical study of stratum corneum, parabasal, and basal layers expressed a strong positive reaction in the perinuclear and cytoplasmic p53 protein. These accumulations of p53 in the cellular cytoplasm occurred due to some considerable pathway. This pathway damages the p53 protein function to prevent unprepared cell proliferation (tumor suppressor).

The strong positive reaction of p53 was documented in various types of animal and human cancers [15, 16, 19, 23, 24]. This result is also compatible with that of the previous study that detected p53 cytoplasmic overexpression and perinuclear expression in equine sarcoids induced by BVP [3, 11, 25, 26]. The previous studies reported that epithelial constituents of fibropapilloma showed strong overexpression of p53 that occurred due to various stress factors.

The observations of positive p53 protein expression in the results of the current study might have occurred due to the impaired of the p53 mechanism that happened from the neoplastic transformation effect of bovine papilloma oncogenic virus. The impaired mechanisms cause a transitory increase of the p53 protein and also a buildup of mutated p53 that is hard to degrade or function.

Bovine papillomavirus can provoke the proliferation of dermal and epithelial cells. The balance between cell death and proliferation is adjusted by apoptosis that performs an important task in the homeostasis and development. The disruption of the apoptotic mechanism is the “hallmark” of the tumor [27–30].

Moreover, few studies have stressed on the function of bovine papillomaviruses oncogenes in the development of cancer by disruption of cellular mechanisms and facilitating neoplastic transformation. The cellular nonentity is recognized due to the interaction between the viral oncoproteins and p53 malfunction. Therefore, understanding the bovine papillomavirus mechanism in the development of cutaneous lesions is necessary for tumor molecular investigations [1, 11, 28–30]. The normal skin sections revealed a negative reaction for p53 expression, and this result is in agreement with that of the previous observation by Rogel et al. [31], who mentioned that p53 protein could not be recognized in the normal cell using immunohistochemistry because of its instability and short half-life. Dissimilarly, strong p53 overexpression was reported in relation with tumor p53 mutation that owns a long half-life. The staining pattern of the p53 overexpression and cytoplasmic sequestration found a critical pathway in the malfunction of the tumor suppressor gene reported in various tumors such as breast cancer and neuroblastomas [25, 32]. Researchers approved that sequestration of wild type p53 into cell cytoplasm is associated with DNA destruction that led to arrest of the cell cycle (G1) and apoptosis in neuroblastoma cell lines [33]. Wang et al. approved the effect of numerous elements in the localization of p53 in the cytoplasm. Jab1 is the element that enables degradation and exclusion of nuclear p53 [34], while Oh et al. found that cytoplasmic anchor protein of p53 was controlled by a Parkin-like ubiquitin ligase (Parc) [35, 36].

In this study, the fibropapilloma or papilloma was investigated macroscopically, microscopically, and immunohistochemically in 3 sheep and 4 goats. This result investigated the function of papillomavirus in the development of tumor in small ruminant as described previously in bovine and humans [3, 37]. These results are compatible with the previous findings of another study on ovine and caprine papillomavirus reported elsewhere in the world [36–40]. Papillomaviruses have approved their essential contribution to the development of human and animal cancers such as skin cancer [1, 3, 41, 42]. Nonetheless, the involvement of papillomaviruses in small ruminants (sheep and goats) is scarce reported in the literature [31, 33, 34].

In this study, the application of immunohistochemistry technique revealed a significant importance in the diagnosis of papillomavirus as well as overexpression of p53 protein, and these results are in agreement with the previous studies [1, 11, 28, 29, 37].

In conclusion, this study showed the clinical and histopathological changes of papillomavirus in Iraqi ruminants. Positive p53 and papillomavirus antigen were determined in the affected animals. The authors advise other future studies to enhance the understanding of the role of p53 protein related to bovine papillomavirus. Also, the relation between different animals papillomavirus needs to be achieved using new molecular tools. Field treatment and control plan should be established to reduce the number of affected animals.

## Figures and Tables

**Table 1:** The details of the papillomavirus feature in the affected animals.

No.	Animal species	Sex	Age	Anatomical locations of the lesions
1	Bovine	Female	5 years	Perianal external genital, chest, abdomen, eyes, face, udder
2	Bovine	Female	4 years	Face, shoulder, udder
3	Bovine	Male	6 months	Face, around eyes, chest, testes
4	Goat	Female	2 years	Udder, lips, eyes
5	Goat	Female	5 years	Lower lips
6	Goat	Female	4 years	External genital
7	Sheep	Female	4 years	Sternum, shoulder, and perianal
8	Sheep	Female	3 years	Chest, perianal
9	Sheep	Female	4 years	Chest, sternum
10	Sheep	Female	5 years	Sternum, perianal

## Data Availability

The data used to support the findings of this study can be made available from the corresponding author upon request. Moreover, the data will be sent to anyone based on two conditions: upon use of the data, the authors should be approved, and also the paper needs to be cited. An agreement in the aforesaid condition will be done if agreed upon.
